# Accounting for complexity – Intervention design in the context of studying social accountability for reproductive health

**DOI:** 10.12688/gatesopenres.13260.1

**Published:** 2021-07-22

**Authors:** Heather McMullen, Victoria Boydell, Joanna Paula Cordero, Petrus S. Steyn, James Kiarie, Patrick Kinemo, Alice Monyo, Mary Awelana Addah, Jacob Tetteh Ahuno, Osei-Bonsu Gyamfi

**Affiliations:** 1 Centre for Global Health, Institute of Population Health Sciences, Queen Mary, University of London, London, E1 4NS, UK; 2 Global Health Centre, Geneva Institute of International and Development Studies, Geneva, 1211, Switzerland; 3 UNDP/UNFPA/UNICEF/WHO/World Bank Special Programme of Research, Development and Research Training in Human Reproduction (HRP Research), Department of Sexual and Reproductive Health and Research, World Health Organization, Geneva, 1202, Switzerland; 4 Sikika, Dar es Salaam, Tanzania; 5 Ghana Integrity Initiative, Accra, Ghana

**Keywords:** Social Accountability, Reproductive Health, Complex Interventions

## Abstract

**Background**: Social accountability interventions aim to propel change by raising community voices and holding duty bearers accountable for delivering on rights and entitlements. Evidence on the role of such interventions for improving community health outcomes is steadily emerging, including for sexual and reproductive health and rights (SRHR). However, these interventions are complex social processes with numerous actors, multiple components, and a highly influential local context. Unsurprisingly, determining the mechanisms of change and what outcomes may be transferable to other similar settings can be a challenge. We report our methodological considerations to account for complexity in a social accountability intervention exploring contraceptive uptake and use in Ghana and Tanzania.

**Main Body**: The Community and Provider driven Social Accountability Intervention (CaPSAI) study explores the relationship between a health facility-focused social accountability intervention and contraceptive service provision in two countries. This 24-month mixed-method quasi-experimental study, using an interrupted time series with a parallel control group, is being undertaken in 16 sites across Ghana and Tanzania in collaboration with local research and implementation partners. The primary outcomes include changes in contraceptive uptake and use. We also measure outcomes related to current social accountability theories of change and undertake a process evaluation.

We present three design features: co-design, ‘conceptual’ fidelity, and how we aim to track the intervention as ‘intended vs. implemented’ to explore how the intervention could be responsive to the embedded routines, local contextual realities, and the processual nature of the social accountability intervention.

**Conclusions**: Through a discussion of these design features and their rationale, we conclude by suggesting approaches to intervention design that may go some way in responding to recent challenges in accounting for social accountability interventions, bearing relevance for evaluating health system interventions.

## Abbreviations

CaPSAI – Community and Provider driven Social Accountability Intervention

HRP - UNDP/UNFPA/UNICEF/WHO/World Bank Special Programme of Research, Development and Research Training in Human Reproduction (HRP Research)

SRHR – Sexual and reproductive health and research

WHO – World Health Organization

## Disclaimer

The authors alone are responsible for the views expressed in this article and they do not necessarily represent the views, decisions or policies of the institutions with which they are affiliated.

## Introduction

The importance of taking a complexity approach to evaluating ‘real world’ health interventions has now been well established ( Craig *et al*., 2013; Greenhalgh & Papoutsi, 2018; Moore *et al*., 2015; Portela *et al*., 2019). Social accountability interventions, which have gained recognition as a part of health systems strengthening and raising community voice, are associated with an increasing range of health benefits ( Kruk *et al*., 2018; Schaaf *et al*., 2017; Van Belle *et al*., 2018). As a result, there is growing interest in how to understand, assess, and scale successful results. We explore the design of a social accountability intervention evaluated in a two-country study aiming to improve quality of care in order to increase contraceptive uptake and use and present three design features that responded to and aimed to account for complexity.

 Social accountability interventions aim to propel community-driven change and empower citizens and communities to hold duty bearers accountable for promised rights and entitlements ( Joshi, 2017). These interventions aim to be community-owned and led and improve life for local citizens by raising their voices, representing their interests, and increasing their capabilities, ultimately transforming power relations. Accountability in the context of sexual and reproductive health and rights has been described as ‘the appropriate prioritisation of sexual and reproductive health and rights (SRHR) and its implementation throughout the health system and ensuring access to SRHR services, with attention to high-quality and respectful care.’ ( Boydell *et al*., 2019). Social accountability is conceptualised as able to bring about change through a series of activities over time. These may include community education and empowerment, increasing the understanding of rights and entitlements, community mobilisation and data collection, a process of evaluation and measurement against standards and priorities, and a process of interfacing duty bearers and rights-holders or service users working to hold them to account. The process of interfacing can be in the form of meetings, public hearings, or other forums where community members can interface with power holders and each other to share concerns, apply pressure, and track change. Social accountability is an ongoing contingent and often political process, and it operates both within and outside of formal structures and processes. It is, amongst other things, evidently complex. However, how social accountability interventions work, whether the theories of change are accurate, and what the key ingredients for success are across contexts and health topics require more empirical insight, particularly as such interventions are taken on by mainstream health actors and implementers across a range of settings.

 A recent World Health Organization (WHO) convened supplement on complexity approaches, and public health guidance describes the multiple component nature, nonlinear causal pathways, role of local context, and general under examination of outcomes as particular challenges for health interventions with multiple priorities and limited resources ( Portela *et al*., 2019). Updated guidance on implementation studies from the Medical Research Council was described by Greenhalgh and Papoutsi as emphasising ‘‘‘non-linearity and iterative local tailoring’ and placed substantially more emphasis on the need for non-experimental, mixed methods and process-based approaches for studying such phenomena’ ( Greenhalgh & Papoutsi, 2018; Moore *et al*., 2015). A WHO department of sexual and reproductive health and research convened community of practice on measuring and evaluating social accountability interventions for reproductive, maternal, and child health reported similar considerations with specific emphasis on power relations and the political nature of accountability interventions ( Boydell *et al*., 2019). 

Here we explore the Community and Provider driven Social Accountability Intervention (CaPSAI), a study that explores the impact of a social accountability process on contraceptive uptake and use ( Steyn *et al*., 2020). It is one of the first studies to evaluate the effectiveness of social accountability interventions on behaviour related to family planning. It aims to build the evidence base on the potential for such interventions to improve SRHR. Literature and evidence on accountability strategies to improve SRHR is steadily emerging ( Boydell *et al*., 2019; Gullo *et al*., 2017; Van Belle *et al*., 2018) though challenges remain in understanding how best to evaluate these programmes and determine best practices for scale-up. Considering the social accountability process as a complex intervention, with numerous actors, a highly influential local context, and a number of interacting components, as well as being a process over time, it was considered essential to move beyond simple intervention thinking and call on complexity approaches to design and evaluate the CaPSAI study. This paper explores how the CaPSAI intervention was designed to respond to real-life conditions and how recent thinking in implementation science and complex intervention evaluation were considered in developing the CaPSAI intervention. We consider three design features and how they aimed to account for complexity; intervention fidelity, co-design, and the intervention as ‘intended vs. implemented.’

## The Community and Provider driven Social Accountability Intervention (CaPSAI)

The Community and Provider driven Social Accountability Intervention (CaPSAI) study aims to make a robust addition to the literature and evidence base on participatory and social accountability processes for health. The CaPSAI Project has been registered at Australian New Zealand Clinical Trials Registry ( ACTRN12619000378123, 11/03/2019). CaPSAI is a quasi-experimental, mixed methods evaluation implemented across 16 sites in Ghana and Tanzania. The intervention was delivered and evaluated by local civil society partners and research organisations over a period of 24 months. Specifically, it explores the role of such interventions for aspects of SRHR by evaluating the impact and process of implementation of an eight-step social accountability intervention on contraceptive uptake and use in low resource settings with low modern contraceptive uptake ( Moore *et al*., 2015). The CaPSAI study aims to describe and examine how social accountability processes are implemented and operationalised, focusing on behaviours, decision-making processes, and the barriers and facilitators of change. The findings aim to be generalisable to other like settings. It also aims to develop more responsive quantitative measures for social accountability interventions and demonstrate the relationship between social accountability processes and the uptake and use of contraceptives and other family planning behaviours (See Steyn *et al*., 2020 for details on the research protocol). 

Contraceptive uptake is evaluated through an interrupted time series design with a control group (ITS-CG). A cohort of women who are new users of contraception is tracked using standardised interview questions across both intervention and control facilities to measure changes in behaviours around contraceptive use over one year. To capture social accountability intermediate outcomes, such as empowerment of women and health providers and expansion of negotiated spaces, a cross-sectional survey using accountability-related psychometric scales is conducted at pre- and post-intervention phases. The effects of the social accountability intervention and the implementation process are measured through a process evaluation comprising context mapping, qualitative interviews, document review, and implementation tracking. Case studies of change are also collected. A process evaluation was seen as essential due to the complex and processual nature of the social accountability intervention and the challenges in determining causal chains and clearly attributing outcomes to intervention inputs ( Moore *et al*., 2015; Palmer *et al*., 2016).

**Figure 1.**
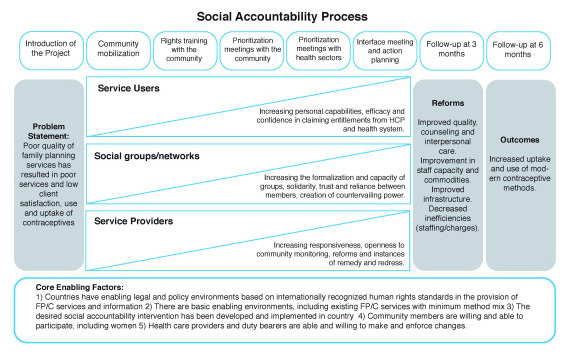
CaPSAI Theory of Change (This figure has been reproduced with permission from Steyn *et al*., 2020).

Figure 1 outlines the CaPSAI theory of change, and Table 1 describes the eight identified steps in more detail. While these are referred to as ‘steps,’ they may be better conceptualised as phases and may contain a number of activities or ranges of activities within each step. We also acknowledge that social accountability interventions are best considered as a process and not as linear discrete steps or tools that will necessarily combine to create social change; however, these steps present a structure for the intervention.

**Table 1.** Standard steps in the CaPSAI social accountability process.

Step	Description
**1.Introduction of the intervention to the** **community**	The implementation partner (usually a civil society organization) meets with local leaders, identifies stakeholders and sets up the infrastructure to deliver the community scorecard.
**2.Mobilization of participants for the** **intervention**	The implementing partner will gather community partners, service providers and the user groups of the health facility/ services to describe the intervention. The implementing civil society organisation should also identify a respected community mobilizer who will jointly facilitate the roll out of the intervention and join the implementation team.
**3.Health, rights and civic education with ** **community participants**	The implementation partner shares information on existing service standards and provides training on rights, good governance and accountability. The group begins to rate existing services against rights-based standards and generate discussion about local priorities.
**4.Prioritization meeting with community**	The implementation partner distills themes and priorities raised by the community. The community groups then collectively score the issues and indicators and set priority areas for action.
**5.Prioritization meeting with duty bearers**	The implementation partner distills themes and priorities raised by the service providers. The providers then collectively score the issues and indicators and set priority areas for action.
**6.Interface meeting and joint action** **planning**	The implementation partner then holds a joint meeting between the community, the service providers and other duty bearers. Following the presentation of results from the prioritization meetings the community groups and the service providers will aim to reach consensus on the ranking of the priority items and the actions required to address them. An action plan with assigned roles and responsibilities will be developed for the following 6–12 month period.
**7.First follow-up meeting with community** **and duty bearers at three months**	Priority areas and action items will be followed up with both the community and service providers. For any unresolved issues these meetings present an opportunity to involve high-level duty bearers of third party pressure (media/ politicians) to increase the pressure to act.
**8.Second follow-up meeting with** **community duty bearers at six months**	A second follow up meeting will enable the monitoring of longer range outcomes and on the remedy of unresolved issues raised in the first follow up meeting. The community and service providers will continue to monitor the action plan for changes in relation to agreed priority areas.

## Key design features in enabling and accounting for local adaptation

The social accountability process is deeply situated in, and contingent upon, the local context and involves multiple actors and factors that can be difficult to account for, inevitably presenting challenges for evaluation. Each process will be and should be different as it responds to locally determined concerns and power relations. As social accountability interventions are not singular discrete interventions, obtaining mainstream achievements and measurements of fidelity, dose, and reach and ensuring knowledge of the ‘active ingredients that allowed the outcomes to take hold can be particularly challenging ( Boydell *et al*., 2019; Craig *et al*., 2013; Moore *et al*., 2015) Thus, in designing the intervention and its evaluation the team considered emerging research and best practice on complex intervention design and evaluation. Table 2 describes dimensions of complexity present in the CaPSAI intervention. We now present three broad considerations: co-design, considering ‘conceptual fidelity’ versus standardisation, and accounting for the intervention as intended versus implemented.

**Table 2.** Accounting for complexity in the Community and Provider driven Social Accountability Intervention (CaPSAI) study design (This table has been reproduced with permission from Steyn *et al*., 2020).

Dimension of complexity	CaPSAI dimension	MRC recommended design features	CaPSAI study design features
A large number of interactions between components within the intervention ( Craig *et al*., 2008).	CaPSAI intervention requires separate and joint activities with community organizations, health providers, and duty bearers to produce an effective space for collective action and change.	A theoretical understanding of how the intervention causes change ( Craig *et al*., 2008).	CaPSAI developed a theory of change ( Figure 1)
A number of behaviors required by those delivering or receiving the intervention ( Craig *et al*., 2008).	Behavior change in varying degrees on the part of community members, health providers, and duty bearers is required for effects to take hold.	A process evaluation design to study the implementation process to address ‘how’ the intervention worked in practice and better understand the ‘active ingredients’ ( Craig *et al*., 2008; Moore *et al*., 2015).	Process evaluation is a main component of the study design
A number of groups or organizational levels targeted by the intervention ( Craig *et al*., 2008).	CaPSAI intervention targets community members, health care providers and duty-bearers at the community and facility level.	A larger sample size and cluster rather than individual level designs ( Craig *et al*., 2008).	Evaluation and sampling at both the community (individual) and health facility level (cluster).
Numerous and variable outcomes ( Craig *et al*., 2008).	The primary outcomes include an increase in contraceptive uptake and indicators of contraceptive use alongside intermediary outcomes such as increases in social capital, collective efficacy, and empowerment.	Use of a range and mix of measures and methods to capture complexity and unintended consequences ( Craig *et al*., 2008; Moore *et al*., 2015).	A range of methods and instruments aim to capture the primary and intermediary outcomes as well as the implementation process itself.
A degree of flexibility or tailoring of the intervention is permitted ( Craig *et al*., 2008).	Implementation should maintain conceptual fidelity to the eight standard steps but allows for local adaptation to produce the effects.	Fidelity should be considered ‘functionally rather than Compositionally ( Fox, 2015) to allow interventions to be responsive to context while still being meaningfully evaluated ( Craig *et al*., 2008; McMullen *et al*., 2015; Moore *et al*., 2015).	The process evaluation and combination of research instruments have been designed to capture functional fidelity in the implementation

### Co-design

Alongside the growth of implementation science has been calls for greater uptake of co-design approaches, particularly in the context of complex interventions ( Craig *et al*., 2013; Moore *et al*., 2015). Co-design stresses equal participation, particularly of ‘end users’, and recognises that interventions will be more responsive and likely to deliver meaningful results if all those with a stake in their delivery and outcomes are involved in all aspects of intervention design, delivery, and evaluation ( Donetto *et al*., 2015; Goodyear Smith *et al*., 2015; Slattery *et al*., 2020). For the CaPSAI study, local civil society organisations with experience in delivering health-related social accountability interventions were selected as implementing partners. Implementing partners then selected community representatives to become members of the implementation and research teams. Community members were also tasked with the facilitation and implementation of the intervention across the 16 sites. In some cases, these community members had already worked with the civil society organisations; in others, they were newly recruited. In order to recognise the wealth of experience and established routines and practices of implementing partners as well as respond to the local context, the intervention was co-designed by the study implementation leads and civil society implementing partners from the local community.

### Stages in the intervention design

Stage 1:      The first stage in the design of the intervention was a review of existing literature and programmes related to social accountability and health. Programme descriptions, evidence, and programme reports for health-related social accountability interventions such as community scorecards, report cards, citizen voice, accountability, and citizen hearings were gathered to define the key phases in the social accountability process (See Table 3). These data were brought together with the findings from the formative phase study UPTAKE Project ( Cordero *et al*., 2019; Steyn *et al*., 2016) and emerging findings from the Evidence Project studies on social accountability in the context of family planning ( Boydell *et al*., 2018) to set the groundwork and proof of concept for CaPSAI and were used to develop the overarching theory of change. From a review, a composite of components (‘steps’) were determined that are typical of social accountability interventions and theories of change (see Table 3. These ‘steps’ structured the second phase of the intervention design process.

**Table 3.** Toolkits reviewed to develop the intervention.

Document Title	Author	Date of publication	Source
**Information and collective action in** **community-based monitoring of schools:** **Field and lab experimental evidence from** **Uganda**	Abigail Barr, F. Mugisha, P. Serneels, A. Zeitlin	Aug.12	https://pdfs.semanticscholar.org/99f5/806ab361f3308d652b9549b390e4f183b672.pdf
**Enhancing governance and health system ** **accountability for people centered** ** healthcare: an exploratory study of ** **community scorecards in Afghanistan**	Edward Anbrasi, Kojo Osei-Bonsu, Casey Branchini, Temor Shah Targhal, Said Habib, Arwal A.	Jul.15	http://bmchealthservres.biomedcentral.com/articles/10.1186/s12913-015-0946-5
**When is community based monitoring** ** effective? Evidence from a randomized** ** experiment in primary health in Uganda**	Bjorkman, M. and Svensson, J.	Apr.10	www.jstor.org.ezproxy.library.qmul.ac.uk/stable/pdf/40601246.pdf
**Scorecards and social accountability for ** **improved maternal and newborn health** ** services: A pilot in the Ashanti and Volta** ** regions of Ghana**	Blake, C., Annorbah- Sarpei, N., Ismaila, Y., Clark, S.	Sep.16	http://ac.els-cdn.com/S0020729216304428/1-s2.0-S0020729216304428-main. pdf?_tid=cce78452-faaa-11e6-aa3c-00000aacb361&acdnat=1487952397_ af8e3ccf5825efa4ea70fa08c3318cee
**Citizen Voice and Action**	Jeff Hall	Jul.14	https://www.escr-net.org/node/366893
**East African Community Regional** ** Reproductive Maternal Newborn and Child** ** Health Scorecard**	Elizabeth Muiruri	Nov.14	https://everyone.savethechildren.net/sites/everyone.savethechildren.net/files/ EAC%20RMNCH%20Scorecard.pdf
**Tanzania Community Score Card** ** Transparency and Accountability Project**	Sandra Michaelson	Nov.13	http://saatlas.org/uploads/files/Results_for_Development_SC_Tanzania_CSC_Interim_ Report.pdf
**Scorecard Toolkit: CARE Malawi**	Sarah Gullo	May.13	https://resourcecentre.savethechildren.net/sites/default/files/documents/6800.pdf
**Maternal Health Alliance Malawi**	Sarah Gullo	Nov.14	http://familyplanning.care2share.wikispaces.net/file/view/MHAP_November_Update.pdf
**Role of Social Accountability in Improving** ** Health Outcomes: Overview and Analysis of** ** Selected INGO Experiences**	K.D. Hoffman	Jun.14	http://www.coregroup.org/storage/documents/Resources/Tools/Social_ Accountability_Final_online.pdf
**Citizen Voice and Action (as reviewed by** ** "Role of Social Accountabilty" paper**	K.D. Hoffman	Jun.14	http://www.coregroup.org/storage/documents/Resources/Tools/Social_ Accountability_Final_online.pdf
**Partnership Defined Quality Framework**	K.D. Hoffman	Jun.14	http://www.coregroup.org/storage/documents/Resources/Tools/Social_ Accountability_Final_online.pdf
**CARE's Community Scorecard**	K.D. Hoffman	Jun.14	http://www.coregroup.org/storage/documents/Resources/Tools/Social_ Accountability_Final_online.pdf
**White Ribbon Alliance Social Watch Approach**	K.D. Hoffman	Jun.14	http://www.coregroup.org/storage/documents/Resources/Tools/Social_ Accountability_Final_online.pdf
**White Ribbon Alliance Uganda Participatory ** **Health Facility Assessment**	K.D. Hoffman	Jun.14	http://www.coregroup.org/storage/documents/Resources/Tools/Social_ Accountability_Final_online.pdf
**GOAL Accountability can Transform Health**	K.D. Hoffman	Jun.14	http://www.coregroup.org/storage/documents/Resources/Tools/Social_ Accountability_Final_online.pdf
**The community scorecard process: ** **methodology, use, successes, challenges,** ** and opportunities**	Jephter Mwanza and Nina Ghambi	Dec.11	http://pubs.iied.org/pdfs/G03207.pdf
**Effects of CARE’s Community Score Card on** ** reproductive health-related outcomes**	Sara Gullo, Christine Galavotti, Anne Sebert Kuhlmann, Thumbiko Msiska, Phil Hastings, C. Nathan Marti	Feb.17	http://journals.plos.org/plosone/article/file?id=10.1371/journal.pone.0171316&type= printable
**Social Accountability Services: Case Study** ** 1: Andhra Pradesh, India: Improving Health ** **Services through Community Score Cards**	Vivek Mistra, P. Ramasankar, Lakshmi Durga, Sanjay Agarwal, J.V.R. Murty	Aug.07	http://unpan1.un.org/intradoc/groups/public/documents/un-dpadm/unpan044598.pdf
**Governance and Transparency Fund, ** **Environmental Governance, Ghana, the ** **Community Scorecard Approach**	Governance and Transparency Programme	2014	https://washmatters.wateraid.org
**Evaluation and Design of Social** ** Accountability Component of the Protection** ** of Basic Services Project, Ethiopia**	Samuel Taddesse, Biraj Swain, Merga Afeta, Gadissa Bultosa	Jun.10	http://siteresources.worldbank.org/INTPCENG/Resources/CSC+Gambia.pdf
**Transparency for development baseline** ** report, Tanzania**	Jean Arkedis, Jessica Creighton, Archon Fung, Stephen Kosack, Dan Levy, Rohit Naimpally, Lindsey Roots and Courtney Tolmie	Sep.16	http://t4d.ash.harvard.edu/sites/default/files/file-uploads/Full%20T4D%20Baseline%2 0Report%20FINAL.pdf
**Accountability in Local Service Delivery: The** ** Tuungane Community Scorecard Approach**	Guillaume Labrecque and Isatou Batonon	May.15	https://www.rescue.org/sites/default/files/document/660/ accountabilityinlocalservicedeliveryenglishfinal.pdf

Stage 2:      For each of the eight social accountability ‘steps’ a set of questions was developed that aimed to elicit the routine practices, knowledge, experiences, and concerns of the two national non-governmental organisations implementing the activities. If routines are highly contextual and specific practices that are ‘recurrent, collective and interactive behaviour patterns’ help implementers structure and make sense of their roles and worlds, it was essential to understand existing routines and roles and the ‘normal practice’ of implementation ( Greenhalgh, 2008, p1269). The key questions aimed to ascertain the ways in which the implementing partners are already addressing the core aspects of intervention fidelity (as considered functionally ( Hawe *et al*., 2004)) in their regular practice and existing implementation strategies for existing social accountability programmes. Understanding these practices in advance allows for designing an intervention that better reflects what may take place in ‘actual’ implementation by not suggesting new or changed practices where they are not necessary. Findings from the first and second design stages were synthesised into a guide for design stages three and four.

Stage 3:      In the third stage of the intervention design, multiple meetings were held over a period of months with the implementation teams prior to the start of implementation. Initial meetings introduced the study and the objectives. Subsequent meetings engaged a discussion structured around the findings of the review and the key questions. The tentative theory of change and identified ‘steps’ were used as prompts to discuss and explain previous experiences of the implementing partners in delivering social accountability interventions. Discussions were recorded and loosely transcribed, and notes were fed back to the team and used to ‘build out’ the intervention. Through an iterative process, an intervention manual to use during the study was put together.

The intervention manual is written in a ‘workbook’ style and does not set out an overly prescriptive or standardised intervention to be delivered. The manual sets out the aims and objectives of the study, along with essential information for study conduct and implementation as part of a research project (which was new for the implementation teams). For each of the eight social accountability ‘steps,’ the manual describes how the step is conceptualised within the theory of change and lists key questions and considerations for implementers with examples that emerged from the co-design process. Workbook pages are included for pre and post-implementation. While the study intervention implementation manual was primarily designed by the implementation team, comprised of WHO team members and civil society partners, the site-specific implementation plans involve the local facilitators and community implementers across the 16 sites. The pre-implementation plan is where implementers set out how their plans adhere to the core tenets of the intervention step and respond to key criteria and concerns, essentially how ‘fidelity’ is composed. This plan is then reviewed by the implementing teams with the implementation leads to discuss how it meets the requirements of the study and achieves fidelity. The post-implementation report allows implementers to account for implementation ‘on the day’ and note any deviations from the plan or to remark on exceptional events.

Stage 4:      In the final stages, feedback on the draft manual was gathered, and further refinements were made, and the ‘workbook’ aspect of the manual was further considered. Finally, the teams worked to finalise the planned intervention and agree on a final manual. Training then took place with the local implementing teams over a period of days.

Tracking the intervention was developed to enable and account for local adaptation. As reflected in the design of the implementation manual, fidelity to the theory of change and core aspects of the intervention is key, and how this fidelity is composed is expected to vary. The (re)consideration of fidelity is one of the intervention design features that aims to account for the complex and contextual nature of social accountability interventions and their success.

### Intervention ‘fidelity’

Recent literature acknowledges that adaptive intervention strategies yield more responsive and localised interventions that may respond better to community needs ( Greenhalgh & Papoutsi, 2018). Some thinking in implementation science and complex interventions indicates the value of considering intervention integrity and fidelity functionally rather than compositionally ( Greenhalgh & Papoutsi, 2018; Hawe *et al*., 2004; McMullen *et al*., 2015; Perez *et al*., 2016). In measuring complex interventions alongside quantitative study designs, this conceptualisation of fidelity has been suggested as a potentially more responsive approach to assessing the integrity of interventions. This requires distilling the essential criteria required to have fidelity to the overarching tenets of the intervention but moves away from an overly prescribed and standardised pathway of implementation. Hawe *et al.* (2004) ask what standardisation is in a complex intervention and suggest *‘rather than defining the components of the intervention as standard … what should be defined as standard are the steps in the change process that the elements are purporting to facilitate or the key functions that they are meant to have*’ (pg.1561). The ‘workbook’ style of the implementation plan for each step develops a form of ‘mini’ site-specific protocol for each intervention step, that adheres to the core aspects that support the theory of change while accounting for local context and adaptability ( McMullen *et al*., 2015). The implementation plans and design also respond to the design of the process evaluation. As described by Palmer *et al*. (2016), ‘ *A key challenge is how to find a balance between the fluidity that complexity and process so obviously warrant and the development of process evaluation aims, questions and procedures in advance (pg 2)*.’ 

A complex social intervention such as a social accountability process, implemented by organisations with previous experience and established routines and practices indicates that the intervention as envisaged prior to implementation will differ slightly from actual implementation ‘on the day.’ To account for this reality, a ‘pre-implementation plan and post-implementation report ‘is included as a part of the intervention design, to be completed before and after each step of the social accountability process.

### The intervention ‘as intended’ versus ‘as implemented’

The pre-implementation plans and post-implementation reports form a part of the document review for the process evaluation research team, alongside the qualitative interviews, context mapping, and case studies of change. The reports support an understanding of the dose, reach, and fidelity of the intervention while accounting for and enabling adaptability.

This feature allows the intervention ‘as intended’ and the intervention ‘as implemented’ to be tracked and to account for divergences at the reporting stage. This may allow for a better understanding of the ‘active ingredients’ in the implementation process and for a better description of what took place over the course of the study. This will assist evaluators in assessing whether the theory of change was accurate, what the causal pathways for intervention outcomes may be, and what may be essential for scalability and generalisability. Alongside the pre-implementation plans and post-intervention reports, the process evaluation contains a method where small case studies of change are gathered. Through in-depth qualitative interviews and document review, reported instances of change thought to be attributed to the social accountability intervention are explored. Here researchers can trace the instances of change and gather the accounts of local actors as to how these changes took place, gathering relevant documentation and triangulating interviews as needed. Drawing on ethnographic methods, these combined accounts help develop the picture represented by the quantitative findings with rich accounts of the intervention in action and local perceptions and descriptions of impact. 

## Conclusion

As described by Hawe and Shiell back in 2004, ‘ *reducing a complex system to its component parts amounts to an “irretrievable loss of what makes it a system”’* ( Hawe *et al*., 2004; p. 1562). ‘Real world’ interventions also experience real-world pressures such as budget constraints, tight timelines, international teams, and so on. Trying to incorporate emerging best practice that challenges the status quo can present challenges. What is described in this paper reflects efforts to incorporate some of the relevant guidance to support a complex and political intervention on what is often a controversial and contested set of rights and health behaviours. This is a reflection on the design process, and the results of the study will be reported, upon completion, elsewhere. Co-design of the intervention, a conceptualisation of fidelity that is as open and adaptive as possible, and using ethnographic approaches to track the intervention steps as intended versus as implemented as part of a process evaluation have been how we considered complexity in the intervention design for the CaPSAI study.

## Data availability

No underlying data are associated with this article.
