# Supplementary material for: Accounting for complexity – Intervention design in the context of studying social accountability for reproductive health
Source: Gates Open Res. 2022 May 23;5:107. Originally published 2021 Jul 22. [Version 2] doi: 10.12688/gatesopenres.13260.2 (PMC9352917; doi:10.12688/gatesopenres.13260.2)
Supplement: Peer review history

# Peer review: Reviewer response for version 2

**DOI:** 10.21956/gatesopenres.14906.r32091

**Published:** 2022-08-04

**Authors:** Deborah D. Diliberto

**Affiliations:** 1 Global Health, Faculty of Health Sciences, McMaster University, Hamilton, ON, Canada

I have read the authors' response and I am satisfied with their revisions. I have no further comments to make.

Does the article adequately reference differing views and opinions?

Yes

Are all factual statements correct, and are statements and arguments made adequately supported by citations?

Yes

Is the Open Letter written in accessible language?

Yes

Where applicable, are recommendations and next steps explained clearly for others to follow?

Partly

Is the rationale for the Open Letter provided in sufficient detail?

Yes

Reviewer Expertise:

Design and evaluation of complex health and social interventions in low resource settings; interdisciplinary and mixed methods approaches.

I confirm that I have read this submission and believe that I have an appropriate level of expertise to confirm that it is of an acceptable scientific standard.

# Peer review: Reviewer response for version 2

**DOI:** 10.21956/gatesopenres.14906.r32093

**Published:** 2022-05-25

**Authors:** Jashodhara Dasgupta

**Affiliations:** 1 SAHAYOG, Lucknow, Uttar Pradesh, India; 2 Feminist Policy Collective, Lucknow, Uttar Pradesh, India

I appreciate the attention paid to the earlier comments and the added materials and revisions in the text of the paper. Nonetheless, I regret that a point I have made in the earlier review has apparently not been acknowledged. 

 The revised paper still does not mention how or when the context analysis was undertaken even while it mentions that the process of ‘co-designing’ recognized the ‘wealth of experience’ and tried to ‘respond to the local context’.

 Without analysis of the context as a preliminary step in designing any SRHR intervention, there remains a risk that the strategy adopted may not be the optimal one to address the issue.

 For instance, it is not mentioned whether the current barriers to low usage of modern contraceptives were analyzed at any stage by the team: were they cultural/gender norms, or supply-side constraints/community scarcity or maybe there were some health system challenges in terms of low accountability to the end-users of SRHR services.

 Without analysis of the barriers, or examination of possible alternative strategies to address the issue and its root causes, there is an implicit decision made that social accountability will best serve the purpose towards attaining the intended SRHR outcomes. Yet the paper does not clarify the reasons why this decision was made, or at what stage it was assumed that the social accountability process would be the most effective answer to low usage of modern contraceptives.

 This fundamental limitation is not acknowledged anywhere, which leads this paper to promote the rather risky approach that context analysis, understanding the root causes and considering various alternative/complementary strategies are not really needed. The paper may tacitly indicate that merely following the eight steps (with attention to co-design, fidelity and intention-implementation comparisons) is sufficient to ensure outcomes.

Does the article adequately reference differing views and opinions?

Partly

Are all factual statements correct, and are statements and arguments made adequately supported by citations?

Yes

Is the Open Letter written in accessible language?

Yes

Where applicable, are recommendations and next steps explained clearly for others to follow?

Not applicable

Is the rationale for the Open Letter provided in sufficient detail?

Yes

Reviewer Expertise:

Gender and equity analysis, rights-based approaches, social accountability, maternal health, sexual and reproductive health and rights, choice and adolescent issues, social and political determinants of health, public health systems governance

I confirm that I have read this submission and believe that I have an appropriate level of expertise to confirm that it is of an acceptable scientific standard, however I have significant reservations, as outlined above.

# Response

**Published:** 2022-07-26

**Authors:** Joanna Cordero

**Affiliations:** World Health Organization, Switzerland

**Response: **Apologies for not responding to your comments in the earlier response to comments. The CaPSAI Project was developed in line with the World Health Organization guideline: “Ensuring human rights in the provision of contraceptive information and services”, which identified nine key health and human rights standards that need to be strengthened: non-discrimination, availability, accessibility, acceptability, Quality of Care (QoC), participation, informed decision-making and choice, privacy/confidentiality and accountability (WHO 2014). The project implemented tested social accountability processes to promote community monitoring and social accountability of contraceptive programs. To do this, we identified national civil society organization using social accountability and were already working or wanting to work on sexual and reproductive health.

 We want to clarify that two types of contextual analysis were undertaken as part of the larger study and project.  The first was led by the research team at the start of each project year to better understand the current social accountability and SRHR programmes that were underway in the locations where the project was taking place. The second was undertaken by the national CSOs facilitating the social accountability activities with the community, they undertook an analysis of the SRHR context locally as part of the project preparation work and then this was discussed at the start of the community engagement process.  It was felt that the national partners supporting the social accountability processes were best placed to undertake the analysis of the local and wider barriers to SRHR as the processes have to be responsive to local contexts.  Though many of these barriers are beyond the reach of social accountability processes (which can often be locally bounded if they are not linked up to wider mobilization efforts) and as you point out, this can limit accountability efforts, it was the national partners in conjunction with their community partners who decided what was the most appropriate barriers to tackle based on their analysis.

 Thank you for your careful consideration of our manuscript.

# Peer review: Reviewer response for version 1

**DOI:** 10.21956/gatesopenres.14495.r31154

**Published:** 2021-10-08

**Authors:** Deborah D. Diliberto

**Affiliations:** 1 Global Health, Faculty of Health Sciences, McMaster University, Hamilton, ON, Canada

This open letter reports the methodological considerations for designing and evaluating CaPSAI, a social accountability intervention exploring contraceptive uptake and use in Ghana and Tanzania. While the letter focuses specifically on a social accountability intervention addressing reproductive health and rights, it makes important contributions to the design of complex interventions more generally, and therefore, will be of interest to readers working in complex health and social interventions research.

 The letter addresses an important issue in complex interventions research – how to account for complexity as part of the contingent and emergent links between the intervention, individuals, and society. Given the challenges with representing the intersecting and intertwining processes of designing a complex intervention, I commend the authors for their work on this letter.

 Throughout the letter, the authors emphasize the notion of complexity as contingent. What is missing, however, is an exploration of *why* accounting for complexity in this way is important. The letter would be strengthened with a short explanation of what gets lost or omitted when taking the mainstream notion of intervention as a linear process and a static collection of materials – or alternatively, what is gained when taking a more contingent and context-specific approach to complexity and design.This could be included in the introduction – for example, revising paragraph 4 to provide more explanation of their view/approach to complexity, rather than references that describe acknowledgements of complexity. 

 The letter would also be strengthened with a short description of the intervention itself. There is a heading with a few paragraphs describing the aims of the CaPSAI and evaluation; however, there is no description of the actual intervention.  What is the social accountability mechanism?  Table 1 describes a community scorecard, but no further explanation is given. It is also unclear what aspects of the intervention were decided/set in advance, and what aspects were co-designed. For example, was the community scorecard decided by the implementation team, then the approach to implementation (i.e. the “intervention manual”) co-designed with the implementing partners?     

 Additionally, the letter would be strengthened with more methodological detail about the stages in the intervention design process to understand how, and to what extent, the aims of co-design were achieved. At present, it is not clear who was involved in each intervention design stage and what methods were used. For example, who conducted the reviews in Stage 1? What (participatory?) methods were used to ‘elicit the routine practices…’ (page 6) in Stage 2 and who synthesized findings from Stage 1 and 2?  Who facilitated the meetings in Stage 3 and which team ‘built out’ the intervention (page 6)? This information would also help readers looking towards similar approaches for intervention design.

 Finally, in the section ‘The intervention ‘as intended’ versus ‘as implemented’, the authors suggest their approach “…may allow for a better understanding of the ‘active ingredients’ in the *implementation process*…” (page 10) – my emphasis. This is interesting because it is a departure from the mainstream thinking that focuses on distilling intervention components and disregards how interventions are implemented in situ. This distinction between implementation versus intervention is also touched on somewhat in the section on ‘Intervention ‘fidelity’’, but overall, the nuance of this argument gets lost in the text. The letter would be improved with more clearly articulated discussion of this distinction and what a focus on implementation offers before going into describing how this was achieved.

Does the article adequately reference differing views and opinions?

Yes

Are all factual statements correct, and are statements and arguments made adequately supported by citations?

Yes

Is the Open Letter written in accessible language?

Yes

Where applicable, are recommendations and next steps explained clearly for others to follow?

Partly

Is the rationale for the Open Letter provided in sufficient detail?

Yes

Reviewer Expertise:

Design and evaluation of complex health and social interventions in low resource settings; interdisciplinary and mixed methods approaches.

I confirm that I have read this submission and believe that I have an appropriate level of expertise to confirm that it is of an acceptable scientific standard, however I have significant reservations, as outlined above.

# Response

**Published:** 2022-05-26

**Authors:** Joanna Cordero

**Affiliations:** World Health Organization, Switzerland

Thank you for your comments.  We found them helpful for improving our paper and have made a number of changes.  These changes are listed below. 

**REVIEWER 3:**

 EDIT 10:

 Throughout the letter, the authors emphasize the notion of complexity as contingent. What is missing, however, is an exploration of why accounting for complexity in this way is important. The letter would be strengthened with a short explanation of what gets lost or omitted when taking the mainstream notion of intervention as a linear process and a static collection of materials – or alternatively, what is gained when taking a more contingent and context-specific approach to complexity and design.This could be included in the introduction – for example, revising paragraph 4 to provide more explanation of their view/approach to complexity, rather than references that describe acknowledgements of complexity.

**Response: **We have added a paragraph on the CaPSAI approach to social accountability and why this is important under the section describing the project.

 EDIT 11:

 The letter would also be strengthened with a short description of the intervention itself. There is a heading with a few paragraphs describing the aims of the CaPSAI and evaluation; however, there is no description of the actual intervention.  What is the social accountability mechanism?  Table 1 describes a community scorecard, but no further explanation is given. It is also unclear what aspects of the intervention were decided/set in advance, and what aspects were co-designed. For example, was the community scorecard decided by the implementation team, then the approach to implementation (i.e. the “intervention manual”) co-designed with the implementing partners?    

**Response:** Thank  you for Reviewers 1 and 3 on their suggestions for fleshing out the stages of intervention design and intervention description. We now refer to the section on Co-design as ‘aspects of co-design’ and have provided more detail where possible on how these aspects of co-design were applied in CaPSAI.  Detail was added on this in the description of Stage 3 of the intervention design including some short examples of how the intervention manual reflects the co-design as well as how the description of the intervention was changed by this. We have also put in a reference to other study resources with more detail on the intervention and the process.

 EDIT 12:

 Additionally, the letter would be strengthened with more methodological detail about the stages in the intervention design process to understand how, and to what extent, the aims of co-design were achieved. At present, it is not clear who was involved in each intervention design stage and what methods were used. For example, who conducted the reviews in Stage 1? What (participatory?) methods were used to ‘elicit the routine practices…’ (page 6) in Stage 2 and who synthesized findings from Stage 1 and 2?  Who facilitated the meetings in Stage 3 and which team ‘built out’ the intervention (page 6)? This information would also help readers looking towards similar approaches for intervention design.

**Response:** As per our response to reviewer 1, EDIT 4, we now refer to the section on Co-design as ‘aspects of co-design’ and have provided more detail where possible on how these aspects of co-design were applied in CaPSAI. 

 EDIT 13:

 Finally, in the section ‘The intervention ‘as intended’ versus ‘as implemented’, the authors suggest their approach “…may allow for a better understanding of the ‘active ingredients’ in the implementation process…” (page 10) – my emphasis. This is interesting because it is a departure from the mainstream thinking that focuses on distilling intervention components and disregards how interventions are implemented in situ. This distinction between implementation versus intervention is also touched on somewhat in the section on ‘Intervention ‘fidelity’’, but overall, the nuance of this argument gets lost in the text. The letter would be improved with more clearly articulated discussion of this distinction and what a focus on implementation offers before going into describing how this was achieved.

**Response:** We address this in the paragraph under the description of CaPSAI on our approach to social accountability.

# Peer review: Reviewer response for version 1

**DOI:** 10.21956/gatesopenres.14495.r31151

**Published:** 2021-10-06

**Authors:** Jashodhara Dasgupta

**Affiliations:** 1 SAHAYOG, Lucknow, Uttar Pradesh, India; 2 Feminist Policy Collective, Lucknow, Uttar Pradesh, India

Summary of the article - it describes a way of designing the implementation and evaluation of the CaPSAI intervention that used Social Accountability methods to improve service uptake for contraceptives. The way it was designed quite deliberately uses complex intervention evaluation as well as implementation science. The three key aspects of the designing that are highlighted in the paper include co-designing, intervention fidelity and tracking the 'intended vs implemented' processes throughout the eight steps of the programme. It is indicated that a separate article will bring out the outcomes and findings of the CaPSAI programme. 

 With regard to my comment about inadequate 'reference to differing views and opinions', my suggestions to improve the paper are as below: 

 Table 1 sets out the steps of the intervention, based on the various programme interventions studied as per Table 3. However the list as indicated in Table 3 is limited to short-duration interventions by international actors/donors/  researchers who set out to make an intervention in an under-resourced setting in the global South. The objective is to improve contraceptive usage, a 'civilizing objective' based on the assumptions that citizen voice and facilitated negotiations with duty bearers will overcome health system challenges, commodity scarcity and cultural/gender  norms. 

 Joshi and Houtzager (2012 ^1^) have usefully made the distinction between 'widgets and watchdogs' and define Social Accountability as 'long-term ongoing political engagement of social actors with the state' as opposed to short term project interventions initiated by researchers or donors that seek to bring about linear processes of change based on the formulaic application of 'steps' of social accountability with insufficient analysis of local context. 

 The paper would have been improved by stating clearly these limitations and referring to this literature that refers to the profoundly political nature of social accountability processes that seek to confront power asymmetries.

 The paper would be strengthened by discussing some of the characteristics of the specific local context: for instance by analyzing whether absence of citizen voice and provider accountability are the only barriers to contraceptive use or whether health system challenges, commodity scarcity and cultural/gender  norms may be at the root of low contraceptive uptake in the area.

 The paper does not sufficiently refer to literature that acknowledges the non-linear and iterative nature of change processes that work within complex socio-political contexts of public-service provision in most under-resourced health systems of LMICs (refer to Schaaf and Dasgupta, 2019 ^2^, Balestra *et al.*, 2018 ^3^, Dasgupta, 2011 ^4^ and so forth).

Does the article adequately reference differing views and opinions?

Partly

Are all factual statements correct, and are statements and arguments made adequately supported by citations?

Yes

Is the Open Letter written in accessible language?

Yes

Where applicable, are recommendations and next steps explained clearly for others to follow?

Not applicable

Is the rationale for the Open Letter provided in sufficient detail?

Yes

Reviewer Expertise:

Gender and equity analysis, rights-based approaches, social accountability, maternal health, sexual and reproductive health and rights, choice and adolescent issues, social and political determinants of health, public health systems governance

I confirm that I have read this submission and believe that I have an appropriate level of expertise to confirm that it is of an acceptable scientific standard, however I have significant reservations, as outlined above.

## References

[1] : Widgets or Watchdogs?. *Public Management Review* .2012;14(2) : 10.1080/14719037.2012.657837 145-162 10.1080/14719037.2012.657837

[2] : "Our fear is finished," but nothing changes: efforts of marginalized women to foment state accountability for maternal health care in a context of low state capacity. *BMC Public Health* .2019;19(1) : 10.1186/s12889-019-7028-2 732 10.1186/s12889-019-7028-2 31185954PMC6560750

[3] : Developing political capabilities with Community-Based Monitoring for health accountability: The case of the Mahila Swasthya Adhikar Manch. *Glob Public Health* .13(12) : 10.1080/17441692.2018.1464586 1853-1864 10.1080/17441692.2018.1464586 29671373

[4] : Ten years of negotiating rights around maternal health in Uttar Pradesh, India. *BMC Int Health Hum Rights* .2011;11 Suppl 3: 10.1186/1472-698X-11-S3-S4 S4 10.1186/1472-698X-11-S3-S4 22376057PMC3287460

# Response

**Published:** 2022-05-26

**Authors:** Joanna Cordero

**Affiliations:** World Health Organization, Switzerland

Thank you for your comments.  We found them helpful for improving our paper and have made a number of changes.  These changes are listed below. 

 EDIT 6:

 Table 1 sets out the steps of the intervention, based on the various programme interventions studied as per Table 3. However the list as indicated in Table 3 is limited to short-duration interventions by international actors/donors/  researchers who set out to make an intervention in an under-resourced setting in the global South. The objective is to improve contraceptive usage, a 'civilizing objective' based on the assumptions that citizen voice and facilitated negotiations with duty bearers will overcome health system challenges, commodity scarcity and cultural/gender  norms.

 EDIT 7:

 Joshi and Houtzager (2012) have usefully made the distinction between 'widgets and watchdogs' and define Social Accountability as 'long-term ongoing political engagement of social actors with the state' as opposed to short term project interventions initiated by researchers or donors that seek to bring about linear processes of change based on the formulaic application of 'steps' of social accountability with insufficient analysis of local context.

 The paper would have been improved by stating clearly these limitations and referring to this literature that refers to the profoundly political nature of social accountability processes that seek to confront power asymmetries.

**Response:  **As per our response to Review 1, EDIT 5, we have added a section under the description of CaPSAI to describe our approach to social accountability.

 EDIT 8:

 The paper would be strengthened by discussing some of the characteristics of the specific local context: for instance by analyzing whether absence of citizen voice and provider accountability are the only barriers to contraceptive use or whether health system challenges, commodity scarcity and cultural/gender  norms may be at the root of low contraceptive uptake in the area.

**Response:** Thank you for this comment. The focus of this paper is describing the methodology of co-desiging the intervention. Other findings from the study, including the context mapping will be reported elsewhere.

 EDIT 9:

 The paper does not sufficiently refer to literature that acknowledges the non-linear and iterative nature of change processes that work within complex socio-political contexts of public-service provision in most under-resourced health systems of LMICs (refer to Schaaf and Dasgupta, 2019; Balestra et al., 2018, Dasgupta, 2011 and so forth).

**Response:** Thank you for Reviewers 2 for their suggestions on addressing the issue of   nonlinearity of social accountability interventions and for directing us to literature. We have added the paragraph under the description of the CaPSAI Project to describe our approach to social accountability and how this fits within the literature on social accountability and included additional references.

# Peer review: Reviewer response for version 1

**DOI:** 10.21956/gatesopenres.14495.r31153

**Published:** 2021-09-17

**Authors:** Stephanie M. Topp

**Affiliations:** 1 College of Public Health, Medical and Veterinary Sciences, James Cook University, Townsville, Qld, Australia

This open letter introduces the CaPSAI (Community and Provider driven Social Accountability Intervention) study which explores the impact of a social accountability process on contraceptive uptake and use in two countries. More specifically, the letter reflects on the design process, which, recognizing that the intervention is a ‘complex social change process’ required specific methodological considerations.

 The letter will be of most interest to readers with closely aligned interests in the design of complex interventions tackling social change processes, and seeking to improve the methodological robustness of such studies. Despite being relatively short it is dense with both conceptual and process-related detail.

 Allowing for that target audience, I found the letter to contain some important and interesting considerations, in particular, the way ‘conceptual fidelity’ was operationalized as a design feature within the study. This is the first time I personally have seen/read about this being done (at least in such explicit terms) and it provides an excellent example of a methodological approach that can clearly help account for complexity within a quasi-experimental study design. The potential for applying this approach to other areas of research where ‘controlling for context’ is inappropriate seem substantial.

 The (preceding) description of *co-design* and later description of *intended vs actual* process tracking felt less novel. Particularly co-design since this is a long-advocated approach to building meaningful and context-specific research (or other endeavors). As the authors note there is now a substantial literature on ‘co-design’ specifically; and there is a long history of participatory approaches including PAR that have incorporated related approaches to research or interventions. Nonetheless, and bearing in mind the likely readership for this letter is one seeking detailed examples of study design, I do see the value in the descriptions provided. 

 There are several areas in which the letter could be strengthened. First, careful editing of the front two sections (Intro / CaPSAI section) to reduce the duplicative mentions of the study’s aims and approach would be worthwhile. A single, clear and precise articulation of the study’s aims (potentially sub-titled for ease of reference) in the Introduction or early in the following CaPSAI sub-section would suffice. There are currently a number of slightly different descriptions (e.g. Intro, para 1, final sentence / Intro para 4, first sentence / CaPSAI, para 1, ‘Specifically…’ ) which affect the clarity of writing and comprehension.

 With regards to the 3 main ‘features’ – consistency of language around the way they are described would also be helpful. Are co-design, conceptual fidelity and accounting for intended vs implemented activities, ‘features’ or ‘considerations’ or ‘approaches’?

 The 4 ^th^ paragraph of the Introduction could be much clearer; rather than long introductions to the sources of information, a focus on the nature of the knowledge/methodological developments in the field of complexity research with reference to the source only via citations would help the reader focus on the key information and not get lost in the names of institutions and journals.

 The Co-design section: I think this would be elevated by having a tighter summary of the 'stages of design' process, in favour of more explicit reflection on how the co-design process was influenced by, and adapted to manage, power dynamics among the many stakeholders, which is an obvious feature of complexity that co-design is, in part, there to address. This might be prompted by responding to the question: how this co-design process differed from a more straightforward, albeit extensive consultation? Notwithstanding that there is the *intention* to recognise local stakeholders' experience as expertise for example, it wasn't clear to me how the co-design stages mitigated the tendency for external experts to dominate decision points. It would be helpful to illustrate examples of adaptations to the study design (away from the original theory of change for example) arising from the co-design process.

 Finally, throughout the letter, there remains a not insubstantial tension between the repeated assertion of the way complexity design must account for context specificity and non-linear pathways on the one hand, and the idea that knowledge arising from these studies will be scalable and generalizable on the other hand. One of the reasons I find the description of ‘conceptual fidelity’ compelling, is precisely because it provides an example of how to plot a methodological path through the epistemological tension created by applying a quasi-experimental study design to a research problem which is contextually contingent. But at a broader level throughout this letter, that tension between describing a study design that produces findings which account for complexity, and the expectation that those findings will be scalable or generalizable does not receive much attention; given the earlier observation that the key readership for this letter is likely to be those with a keen methodological interest, closer reflection on that tension by the authors is recommended.

Does the article adequately reference differing views and opinions?

Yes

Are all factual statements correct, and are statements and arguments made adequately supported by citations?

Yes

Is the Open Letter written in accessible language?

Yes

Where applicable, are recommendations and next steps explained clearly for others to follow?

Partly

Is the rationale for the Open Letter provided in sufficient detail?

Yes

Reviewer Expertise:

Health systems research with a focus on governance, accountability and trust; qualitative methodologies.

I confirm that I have read this submission and believe that I have an appropriate level of expertise to confirm that it is of an acceptable scientific standard, however I have significant reservations, as outlined above.

# Response

**Published:** 2022-05-26

**Authors:** Joanna Cordero

**Affiliations:** World Health Organization, Switzerland

Thank you for your comments.  We found them helpful for improving our paper and have made a number of changes.  These changes are listed below. 

**REVIEWER 1:**

 EDIT 1:

 First, careful editing of the front two sections (Intro / CaPSAI section) to reduce the duplicative mentions of the study’s aims and approach would be worthwhile. A single, clear and precise articulation of the study’s aims (potentially sub-titled for ease of reference) in the Introduction or early in the following CaPSAI sub-section would suffice. There are currently a number of slightly different descriptions (e.g. Intro, para 1, final sentence / Intro para 4, first sentence / CaPSAI, para 1, ‘Specifically…’ ) which affect the clarity of writing and comprehension.

**Response:** We have removed some of the duplication regarding the aims of the CaPSAI intervention from the introduction.

 EDIT 2:

 With regards to the 3 main ‘features’ – consistency of language around the way they are described would also be helpful. Are co-design, conceptual fidelity and accounting for intended vs implemented activities, ‘features’ or ‘considerations’ or ‘approaches’?

**Response: **We have standardised language across the text to refer to the three design components versus features, considerations or approaches. 

 EDIT 3:

 The 4th paragraph of the Introduction could be much clearer; rather than long introductions to the sources of information, a focus on the nature of the knowledge/methodological developments in the field of complexity research with reference to the source only via citations would help the reader focus on the key information and not get lost in the names of institutions and journals.

**Response:** We have removed references to institutions in the Introduction paragraph as suggested, to improve readability.

 EDIT 4:

 The Co-design section: I think this would be elevated by having a tighter summary of the 'stages of design' process, in favour of more explicit reflection on how the co-design process was influenced by, and adapted to manage, power dynamics among the many stakeholders, which is an obvious feature of complexity that co-design is, in part, there to address. This might be prompted by responding to the question: how this co-design process differed from a more straightforward, albeit extensive consultation? Notwithstanding that there is the *intention* to recognise local stakeholders' experience as expertise for example, it wasn't clear to me how the co-design stages mitigated the tendency for external experts to dominate decision points. It would be helpful to illustrate examples of adaptations to the study design (away from the original theory of change for example) arising from the co-design process.

**Response:** Thank you for Reviewers 1 and 3 on their suggestions for fleshing out the stages of intervention design and intervention description. We now refer to the section on Co-design as ‘aspects of co-design’ and have provided more detail where possible on how these aspects of co-design were applied in CaPSAI.  Detail was added on this in the description of Stage 3 of the intervention design including some short examples of how the study intervention manual reflects the co-design as well as how the description of the intervention was changed by this. We have also put in a reference to other study resources with more detail on the intervention and the process.

 EDIT 5:

 Finally, throughout the letter, there remains a not insubstantial tension between the repeated assertion of the way complexity design must account for context specificity and non-linear pathways on the one hand, and the idea that knowledge arising from these studies will be scalable and generalizable on the other hand. One of the reasons I find the description of ‘conceptual fidelity’ compelling, is precisely because it provides an example of how to plot a methodological path through the epistemological tension created by applying a quasi-experimental study design to a research problem which is contextually contingent. But at a broader level throughout this letter, that tension between describing a study design that produces findings which account for complexity, and the expectation that those findings will be scalable or generalizable does not receive much attention; given the earlier observation that the key readership for this letter is likely to be those with a keen methodological interest, closer reflection on that tension by the authors is recommended.

**Response:** Thank you for Reviewers 1 and 2 for their suggestions on addressing the issue of   nonlinearity of social accountability interventions and for directing us to literature.  We have added a paragraph under the CaPSAI study description to describe our approach to social accountability. We acknowledge that there are many approaches to social accountability. On one end, it is viewed as a short-term, bounded tactical intervention and at the opposite end of the continuum are approaches that understand social accountability as a process whereby service users, as citizens, demand their legal rights and contribute to social transformation. CaPSAI aims to bridge the two ends of the continuum by codifying the approach of two national groups that have been at the forefront of transparency and accountability for years into short-term interventions.

 We also added a few lines addressing generalizability in the conclusion.
